# Optical intrinsic signal imaging with optogenetics reveals functional cortico-cortical connectivity at the columnar level in living macaques

**DOI:** 10.1038/s41598-019-42923-2

**Published:** 2019-04-23

**Authors:** Yu Nakamichi, Kai Okubo, Takayuki Sato, Mitsuhiro Hashimoto, Manabu Tanifuji

**Affiliations:** 1 Lab for Integrative Neural Systems, RIKEN Center for Brain Science, Wako, Saitama, 351-0198 Japan; 2 Department of Complexity Science and Engineering, Graduate School of Frontier Sciences, The University of Tokyo, Kashiwa, Chiba, 277-8561 Japan; 3 Department of Neuroanatomy and Embryology, Fukushima Medical University, Fukushima, Fukushima, 960-1295 Japan; 4 Department of Life Science and Medicine, Bio-Science Faculty of Science and Engineering, Waseda University, Shinjuku, Tokyo, 169-8555 Japan

**Keywords:** Striate cortex, Neural circuits, Cognitive neuroscience

## Abstract

Despite extensive research on primate cognitive function, understanding how anatomical connectivity at a neural circuit level relates to information transformation across different cortical areas remains primitive. New technology is needed to visualize inter-areal anatomical connectivity in living monkeys and to tie this directly to neurophysiological function. Here, we developed a novel method to investigate this structure-function relationship, by combining optical intrinsic signal imaging (OISI) with optogenetic stimulation in living monkeys (opto-OISI). The method involves expressing channelrhodophsin-2 in one area (source) followed by optical imaging of optogenetic activations in the other area (target). We successfully demonstrated the potential of the method with interhemispheric columnar projection patterns between V1/V2 border regions. Unlike the combination of optogenetics and functional magnetic resonance imaging (opto-fMRI), opto-OISI has the advantage of enabling us to detect responses of small clusters of neurons, even if the clusters are sparsely distributed. We suggest that opto-OISI can be a powerful approach to understanding cognitive function at the neural circuit level, directly linking inter-areal circuitry to fine-scale structure and function.

## Introduction

The goal of systems neuroscience is not only to identify the functions of the brain, but also to understand the underlying computations^1^. Therefore, it is essential to study function together with the underlying neural circuits, particularly in non-human primates. However, this is challenging in non-human primates for several reasons. First, it requires *in vivo* studies to identify point (source)-to-point (target) connectivity across brain areas separated by up to several centimeters, followed by recordings to investigate mechanisms that shape the response properties of target neurons from the response properties of anatomically connected source neurons. Although various molecular labeling techniques are now available for finding connected pairs *in vivo* in rodents^2^, they generally fail to translate to monkeys because of genetic differences that impede transfection. Second, lack of knowledge about the fine-scale functional structures within cortical areas prevents us from identifying cortico-cortical connections. For example, connected pairs that share the same receptive fields were identified in the early visual areas^3^. This was possible because of the well-known retinotopic maps^4^ in these areas and of an *a priori* assumption that neurons with the same receptive field should be connected. However, functional structures in many cortical areas are not well-defined.

To overcome these challenges, we developed an unbiased technique to map cortico-cortical connections at a columnar spatial resolution in living monkeys (opto-OISI). The opto-OISI is a combination of optical imaging of neural responses (optical intrinsic signal imaging, OISI)^5–7^ and optogenetics^8–13^. We densely and extensively expressed channelrhodopsin-2 (ChR2) in neurons in one cortical area (source area) by adeno-associated viral (AAV) vector. We then applied focal light stimulation to neurons in the source area and used optical imaging at the target area to visualize the evoked neural responses (activation maps in the area receiving the projections). Using OISI, we were able to visualize surface neural activation patterns within a large cortical area (e.g. 1.35 cm^2^ in our system) at columnar spatial resolution. Therefore, scanning the focal light stimulation across the source area enabled us to identify at columnar resolution, without any *a priori* assumptions, unknown cortico-cortical projection patterns between the source and target areas.

Optogenetics is a technique for expressing photosensitive proteins such as ChR2 in neurons, to control neural activity by light irradiation. While electrical micro-stimulation is a conventional technique to focally activate neurons, optogenetics is more advantageous than electrical micro-stimulation because it allows the control of specific types of cells and/or sub-structures in each cell expressing ChR2. In this study, we expressed ChR2 specifically in dendrites and cell soma by using myosin Va binding domain of melanophilin (MBD)^11,12^. This allowed us to avoid stimulating axons of passage, which is difficult to avoid in electrical micro-stimulation.

**Figure 1:**
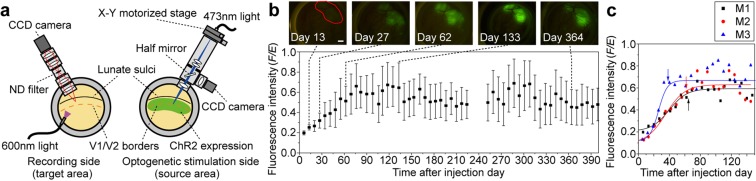
Schematic diagram of the experimental setup (**a**) and time courses of ChR2 expression (**b**,**c**). (**a**) V1/V2 region of both hemispheres was exposed. The right hemisphere (source area) was for injection of AAV9-CaMKIIα-hChR2(ETTC)-EYFP-MBD and focal light stimulation of the expressed ChR2. The left hemisphere (target area) was for optical intrinsic signal imaging (OISI). In the source area, optical apparatus for focal light stimulation was equipped with an X-Y motorized stage for scanning sites of focal light stimulation and a CCD camera for monitoring the locations of the sites relative to the surface vessel pattern. A tandem-lens optical system was used for OISI. The target area was illuminated with 600 nm wavelength of light in OISI. (**b**) Time course of ChR2 expression in the source area (M1). The vertical and horizontal axes represent mean fluorescence intensity and time after the injection, respectively. The error bar indicates standard deviation (see Methods). Five raw fluorescence images are shown and a region enclosed by the red line indicates the AAV9-CaMKIIα-hChR2(ETTC)-EYFP-MBD injection area (Scale bar, 2 mm). (**c**) Time courses of ChR2 expression (mean fluorescence intensity) in three monkeys (M1–M3). The mean fluorescence intensity was fitted with a sigmoidal function (solid line). Arrows indicate 65 (M1), 63 (M2), and 38 (M3) days after AAV9-CaMKIIα-hChR2(ETTC)-EYFP-MBD injection where ChR2 expression reached 90% of the plateau. Please note that the cortical surface of the target area was exposed for OISI 30–70 days after the vector injection when ChR2 expression reached a plateau.

We examined the feasibility of the proposed method for interhemispheric connectivity of V1/V2 border regions (Fig. 1a), building upon previous postmortem anatomical studies that revealed interhemispheric monosynaptic connections through the callosal commissure^14–18^. We expressed ChR2 around the V1/V2 border in the right hemisphere (source area). Several weeks later, we identified cortico-cortical connection patterns by optogenetically stimulating sites at the V1/V2 border in the source area and applying OISI at the V1/V2 border in the left hemisphere (target area). The cortico-cortical connections identified by opto-OISI were validated with electrophysiological recordings.

## Result

We used AAV vectors (serotype 9), AAV9-CaMKIIα-hChR2(ETTC)-EYFP and AAV9-CaMKIIα-hChR2(ETTC)-EYFP-MBD. AAV9-CaMKIIα-hChR2(ETTC)-EYFP expressed a humanized ChR2 (E123T/T159C)^19^ of which the c-terminal was fused to enhanced yellow fluorescence protein [hChR2(ETTC)-EYFP] under the control of the CaMKIIα promoter. AAV9-CaMKIIα-hChR2(ETTC)-EYFP-MBD expressed hChR2(ETTC)-EYFP of which the c-terminal was fused to MBD [hChR2(ETTC)-EYFP-MBD] under the control of the CaMKIIα promoter. We used the CaMKIIα promoter to express hChR2(ETTC)-EYFP and hChR2(ETTC)-EYFP-MBD in excitatory neurons^10,20^. The EYFP-tag visualized the expression and localization of ChR2 in neurons.

We used AAV9-CaMKIIα-hChR2(ETTC)-EYFP-MBD to restrict ChR2 expression to the soma and dendrites of excitatory neurons. AAV9-CaMKIIα-hChR2(ETTC)-EYFP was used as a control to test the specificity of ChR2 expression. We observed the hChR2(ETTC)-EYFP expression in soma, dendrites, and axon of neurons. In contrast, the hChR2(ETTC)-EYFP-MBD expression was considerably well localized to the soma and dendrites because MBD is a sequence involved in intracellular transport mechanisms of expressed proteins (Supplementary Fig. 1)^11,12^.

### Time required for expression of ChR2 in macaques

Since there have been no systematic studies examining the time course of ChR2 expression in macaques, we first examined the time required for ChR2 expression to reach plateau by visualizing EYFP florescence intensity at the cortical surface (Fig. 1b). In monkey 1 (M1), the fluorescence intensity progressively increased and reached a plateau that lasted for more than one year (upper panel). The time required for the fluorescence intensity to reach a plateau was 65 days after the vector injection (lower panel; see Methods and Supplementary Fig. 2 for quantification). The time to reach a plateau was similar in M2 (63 days) but it was earlier in M3 (38 days) (Fig. 1c). Although there were some variations, we conducted OISI experiments more than 50 days after the injection of AAV9-CaMKIIα-hChR2(ETTC)-EYFP-MBD, when fluorescence intensity became reasonably strong and stable.

### OISI with optogenetic stimulation reproducibly identified point-to-point connections between the left and right V1/V2 border regions

To test the feasibility of opto-OISI, we mapped the interhemispheric connections at the V1/V2 border of macaques as a model system (Fig. 1a). Previous anatomical studies revealed that neurons around the V1/V2 border receive projections from the other hemisphere through the corpus callosum^14–18^. This callosal connection between left and right V1/V2 border regions is thought to be essential to guarantee the continuity of left and right visual fields across the vertical meridian, and thus the connections are supposed to be retinotopic^17,18^.

**Figure 2:**
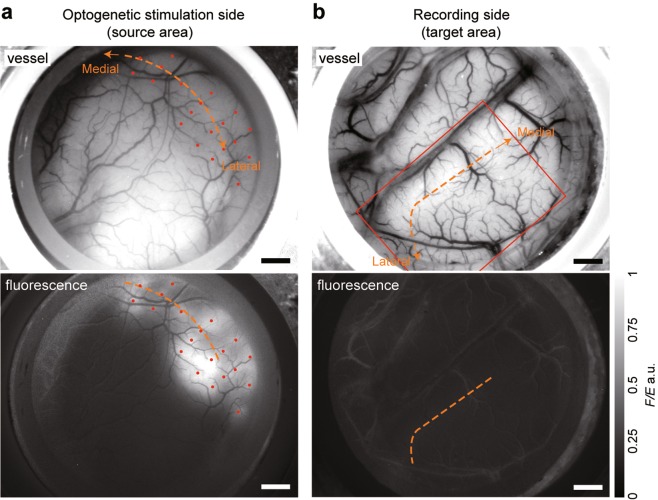
Surface fluorescence patterns revealed that ChR2 was expressed in the source area but not in the target area. (**a**) Vessel pattern (upper panel) and a quantified fluorescence image *F*/*E* (lower panel) of the source area where the optogenetic stimulation was given (right hemisphere). Red dots, AAV9-CaMKIIα-hChR2(ETTC)-EYFP-MBD injection sites. (**b**) Vessel pattern (upper panel) and a quantified fluorescence image *F*/*E* (lower panel) of the target area where optical intrinsic signals were recorded (left hemisphere). Lack of fluorescence in the target area is an indication that ChR2 was not transported through axon. Red square, the OISI imaging area. Dashed orange lines, the V1/V2 borders. All images were obtained 97 days after the vector injection in the source area. Scale bars, 2 mm.

We identified the V1/V2 borders based on the difference in spatial patterns of ocular dominance and/or orientation columns between V1 and V2 (Supplementary Fig. 3). Next, we densely and extensively injected the AAV9-CaMKIIα-hChR2(ETTC)-EYFP-MBD to cover the V1/V2 border in the source area (Fig. 2a, upper panel). After 97 days, ChR2 was expressed in a large portion of the V1/V2 border region with some inhomogeneity in fluorescent intensity (Fig. 2a, lower panel), while we could not observe fluorescence in the V1/V2 border region in the target area at the same scale of fluorescence intensity (Fig. 2b). This result was consistent with the previous *in vitro* study where fusion with MBD localizes expression primarily in dendrites and cell soma (see also Supplementary Fig. 1)^11^.

**Figure 3:**
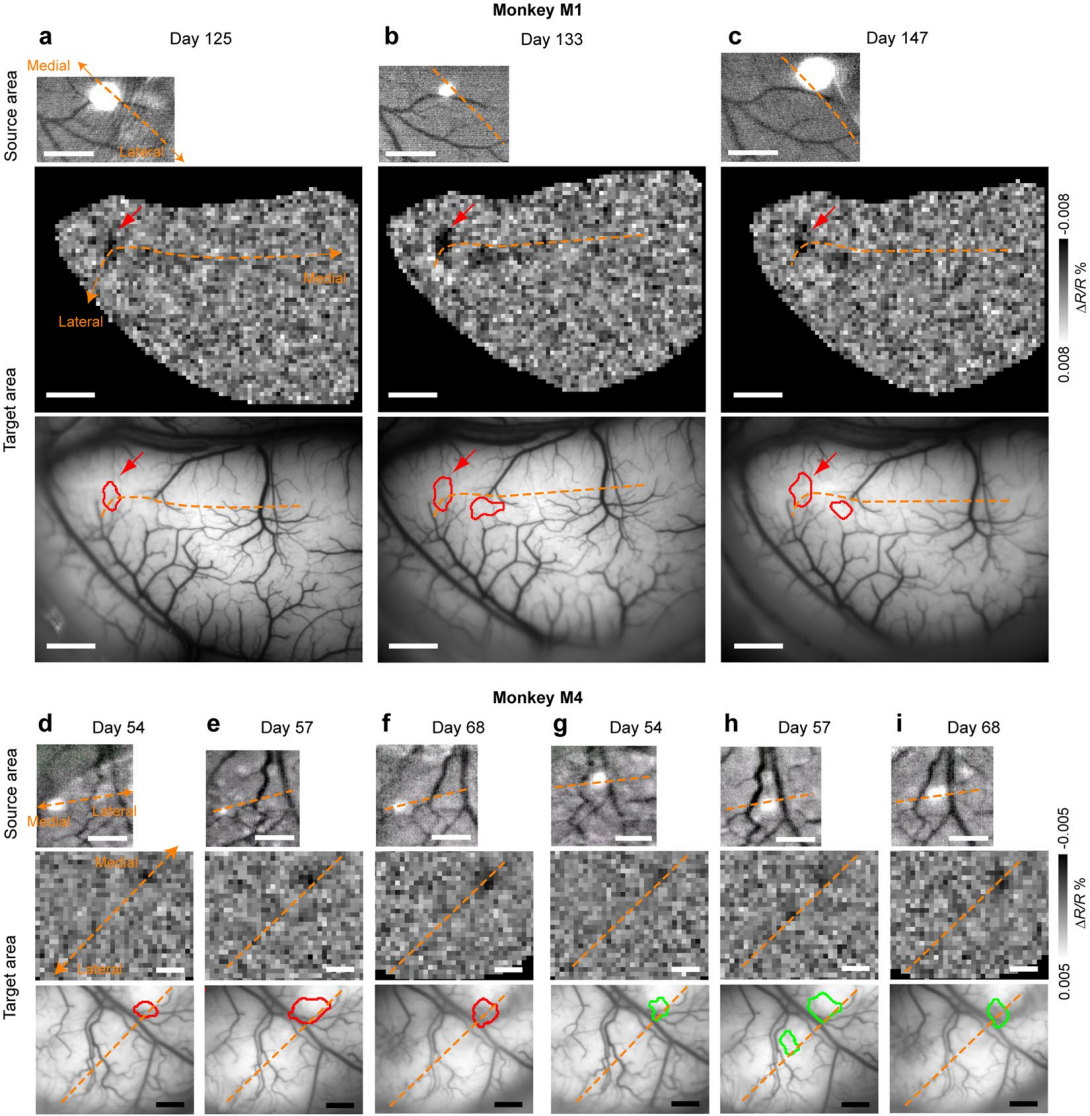
Reproducible patterns of localized optical responses in the target area evoked by a focal optogenetic light stimulation in the source area. (**a**-**c**) Optical responses obtained from M1. Each column shows the site of the optogenetic light stimulation in the source area (top), activation maps elicited by the stimulation in the target area (middle), and activation spots overlaid on the surface vessel patterns (bottom). The activation spots were obtained by demarcating focal regions of darkening in the activation maps (see Methods) as darkening in OISI is known to reflect regions of neural activation. The data were obtained in 125 (**a**), 133 (**b**), and 147 days (**c**) after the AAV9-CaMKIIα-hChR2(ETTC)-EYFP-MBD injection. Relative positions of stimulation sites (top, white spots) and activation spots (bottom) to the surface vessel pattern reveal that optical responses elicited by optogenetic stimulation were reproducible. Scale bars, 1 mm (top) and 2 mm (middle and bottom). Dashed orange lines, the V1/V2 border. (**d**–**i**) Two other cases from a different monkey (M4) showing reproducibility. The data were obtained in 54 (**d**,**g**), 57 (**e**,**h**), and 68 (**f**,**i**) days after the AAV9-CaMKIIα-hChR2(ETTC)-EYFP-MBD injection. The same site was stimulated in (**d**–**f**) and in (**g**–**i**), but the sites between (**d**–**f**) and (**g**–**i**) were different (top, white spots). Scale bars, 1 mm. Other conventions are the same as in (**a**–**c**).

Focal optogenetic stimulation of V1/V2 border of the source area (right hemisphere) evoked a localized optical response in the target area (left hemisphere; Fig. 3). The response was confined to the V1/V2 border region (the region within 1 mm from the border), and the stimulation in V1 away from the V1/V2 border region in the source area did not evoke responses anywhere in the target area (Supplementary Fig. 4). The magnitude of intrinsic signals evoked by focal light stimulation was 6 times smaller than that evoked by whole field visual grating stimuli presented on the visual field (Supplementary Fig. 3). The smaller magnitude is not so surprising because optical responses at the target V1/V2 regions were only elicited by the sub-population of neurons receiving callosal projections.

The observed cortico-cortical projection patterns were reproducible. In Fig. 3a–c (M1), stimulation at the same site on different days (top row) elicited activation at the same location of the V1/V2 border region in the target area (red arrows in middle and bottom rows). Extracted contours of activation spots overlaid on the surface vessel pattern revealed that the activation spots obtained on different days coincided well in location and size (the area on average, 0.880 mm^2^; bottom row). Furthermore, we observed another activation spot in the right that was also near the V1/V2 border region; the spot was reproducible in two out of three recordings (Fig. 3b,c, middle and bottom rows).

Similarly, in a different monkey (M4), focal light stimulation of the V1/V2 border region in the source area on different days evoked consistent localized responses around the V1/V2 border region in the target area (Fig. 3d–i). In Fig. 3d–f, the identical site stimulation (top row) reproducibility evoked an activation spot (middle and bottom rows). In Fig. 3g,h, a site different from Fig. 3d–f (top row) was stimulated, and the stimulation reproducibly elicited an activation spot that was the same spot shown in Fig. 3d–f (bottom row). Therefore, this result revealed convincing evidence for a convergent projection from right (source) to left (target) V1/V2 border region as well (see below).

In summary, these results revealed that connections between the left and right V1/V2 border regions are point-to-point and that the proposed method is feasible to reproducibly identify cortico-cortical projection patterns at a columnar spatial resolution.

### Electrophysiological validation

**Figure 4:**
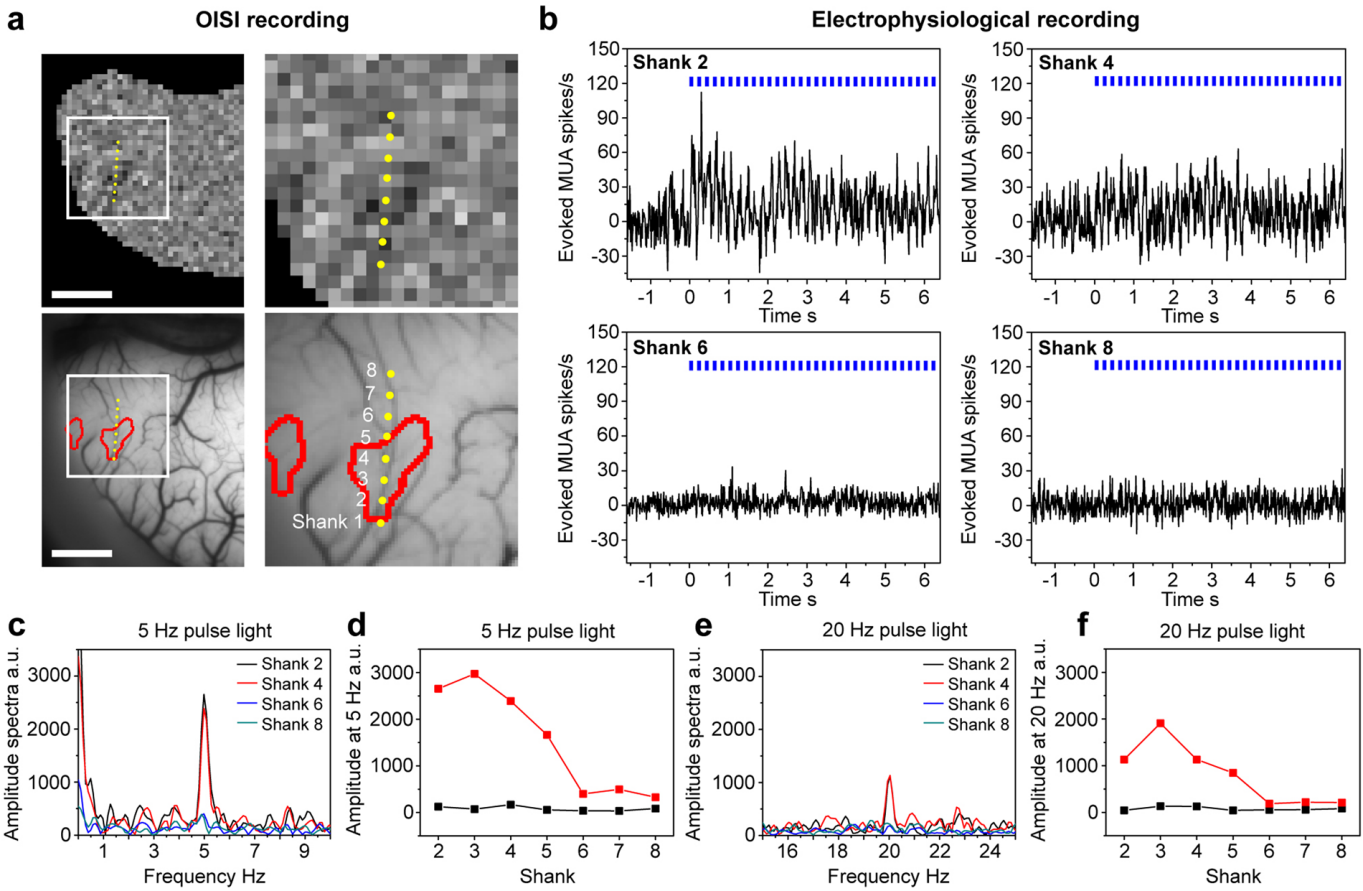
Validation by recording electrophysiological responses around the activation spot in the target area. (**a**) Activation spots elicited by a focal light stimulation. An electrode array was penetrated across one of the spots (yellow dots). Scale bars, 2 mm. The right panels are enlarged views of the demarcated regions in the left. (**b**) PSTHs for the shanks 2, 4, 6, and 8. The location of optogenetic stimulation was the same as in the OISI recording. The stimulus time period was from 0 to 6.4 s (5 Hz pulse, blue squares). In the recording of each shank, the mean spontaneous firing rate was calculated during the pre-stimulus period (−1.6–0 s) and was subtracted from the entire PSTH. Bin size, 10 ms. (**c**) Amplitude spectra for the shanks 2, 4, 6, and 8 calculated in the stimulation period in (**b**) (0–6.4 s). (**d**) Spectral amplitudes at 5 Hz calculated in the stimulation period of 5 Hz pulse light (red) and in the pre-stimulus period (black). (**e**) Amplitude spectra for the shanks 2, 4, 6, and 8 calculated in the stimulation period of 20 Hz pulse light. (**f**) Spectral amplitudes at 20 Hz calculated in the stimulation period of 20 Hz pulse light (red) and in the pre-stimulus period (black). The shank 1 was not available because of cable disconnection.

We validated these opto-OISI cortico-cortical projection patterns via electrophysiological recordings. On a different day after OISI recordings, we recorded multiunit activity (MUA) by penetrating an electrode array across the activation spot (Fig. 4a). We applied light stimulation at the same site as in the preceding OISI recordings. The MUAs recorded from the shanks overlapping the activation spot (shanks 2 to 4) showed elevated firing rate during 5 Hz light stimulation (Fig. 4b, upper panels). In contrast, outside the activation spot (shanks 6 to 8), the firing rate was not modulated by the light stimulation (Fig. 4b, lower panels). Since optogenetic stimulation of ChR2 controls neural firing at a millisecond timescale^8–10^, MUA should be modulated at the frequency of the pulse light stimulation if increase of neuronal firing was elicited by the projection from the source area. In fact, the spectra of the peristimulus time histograms (PSTHs) showed a peak at the pulse light frequency (5 Hz) only if the shanks were inside the activation spot (Fig. 4c). The modulation at 5 Hz was centered at shank 3 during the light stimulation, but not observed in the pre-stimulus period (Fig. 4d). These peaks and modulations were also observed when we used 20 Hz light stimulation (Fig. 4e,f).

**Figure 5:**
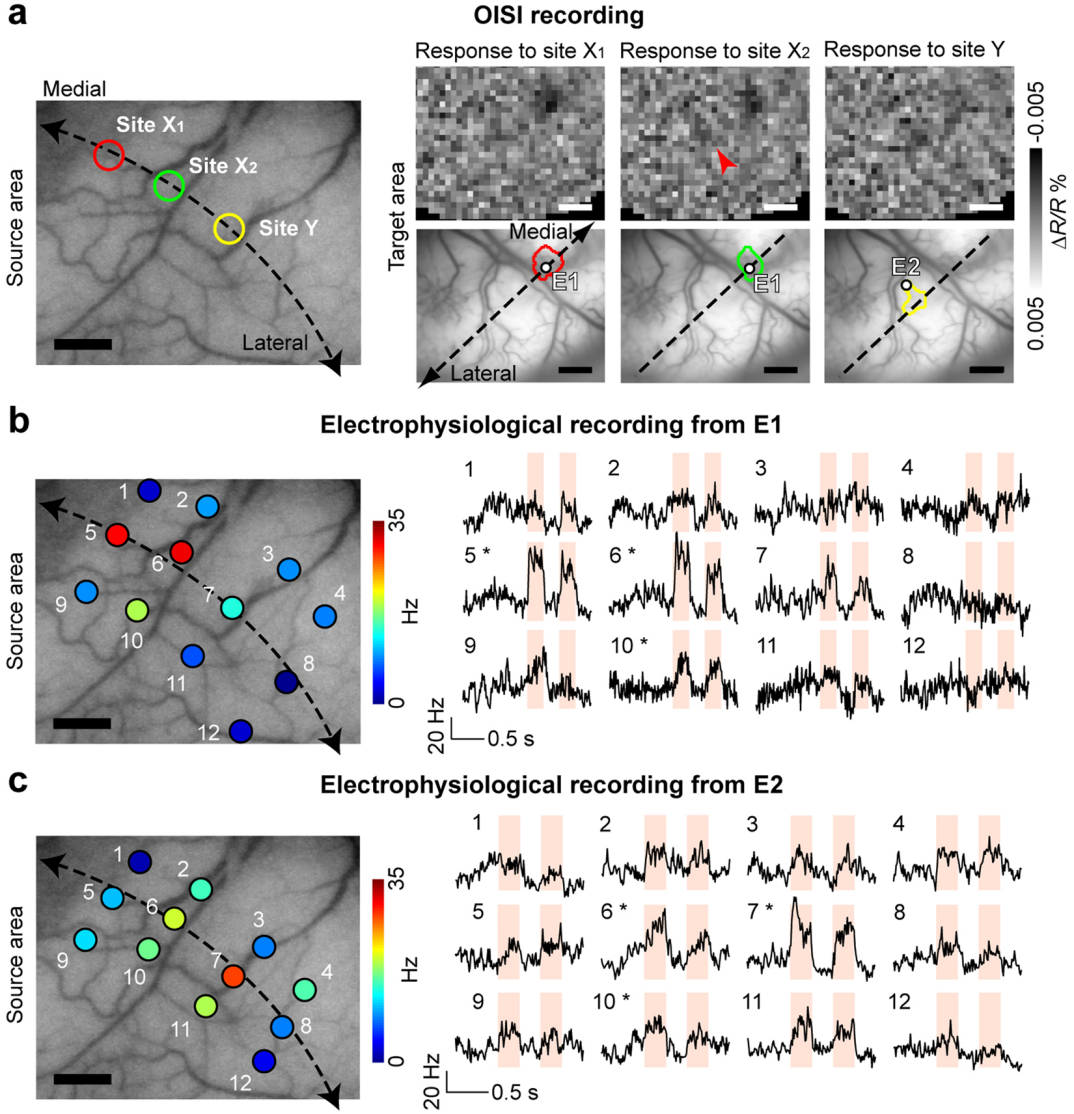
Validation by scanning optogenetic stimulation sites in the source area. (**a**) Activation maps obtained from the preceding OISI. Left panel, sites of optogenetic stimulation (X_1_, X_2_, and Y) overlaid on the surface vessel pattern of the source area. Right panel, activation maps (top) and activation spots (bottom) elicited by stimulation of sites X_1_, X_2_, and Y. The white dots in the activation spots indicate the location of electrodes in subsequent electrophysiological recordings (E1 and E2). The activation spots elicited by stimulation of sites X_1_ and X_2_ are the reproduction of Fig. 3f,i. (**b**,**c**) Electrophysiological responses recorded from E1 (**b**) and E2 (**c**). Left panel, sites of light stimulation in the source area where color indicates mean evoked responses recorded from E1 (**b**) and E2 (**c**). Right panel, PSTHs for stimulation of sites 1–12. The shaded time periods represent periods of 2 Hz pulse optogenetic stimulation. Asterisks, significant modulation of the mean firing rate (p < 0.001, t-test) by optogenetic stimulation (shaded time periods) compared with the mean firing rate at the resting state (0.5 s period before the onset of optogenetic stimulation). Scale bars, 1 mm. Dashed lines, the V1/V2 border.

We also examined whether a localized neural activation was exclusively evoked by optogenetic stimulation at a specific site in the source area in M4. We penetrated an electrode in each of the activation spots in the target area (left hemisphere) and searched for sites in the source area (right hemisphere) where the optogenetic stimulation evoked MUAs (Fig. 5). The preceding OISI results are summarized in Fig. 5a (left panel, the source area for focal optogenetic stimulation; right panels, activation spots in the target area). Focal optogenetic stimulation of sites X_1_ and X_2_ (Fig. 5a, left panel) evoked localized responses at the same spot (Fig. 5a, right panels, red and green contours in the left and middle panels, respectively). As shown already in Fig. 3d–i, the converging projections were reproducible. On the other hand, the focal optogenetic stimulation of site Y evoked localized activation at a spot as shown in Fig. 5a (rightmost panel, yellow contour). Although the response was too weak to draw a contour, we also noticed that site X_2_ stimulation elicited activation at the region corresponding to the spot with the yellow contour (arrowhead in Fig. 5a, upper-middle map in right panels). Then, we recorded MUAs from these two spots by electrodes 1 and 2 (Fig. 5a, right panels, E1 and E2) while stimulating multiple sites, 1 to 12, in the source area (Fig. 5b,c, left panels). The recordings from E1 showed that MUA was strongly modulated by the pulse light stimulation applied to sites 5 and 6 as expected from OISI (Fig. 5a,b). Although there was weak but significant modulation by stimulation of site 10, there was no activation elicited by the rest of sites (Fig. 5b). On the other hand, the recordings from E2 showed that MUA was modulated by the pulse light stimulation given to sites 6 and 7 (Fig. 5c). Therefore, the results of MUA recordings from E2 were also consistent with the preceding OISI. There was significant modulation by stimulation of site 10 both in E1 and E2. MUA responses to site 10 stimulation could not be used to confirm OISI results because we did not examine site 10 stimulation in the preceding OISI recording.

Thus, opto-OISI faithfully maps the modulation of postsynaptic action potentials in the target area, reflecting interhemispheric projections from the source area.

### Callosal connection patterns around the V1/V2 border region

**Figure 6:**
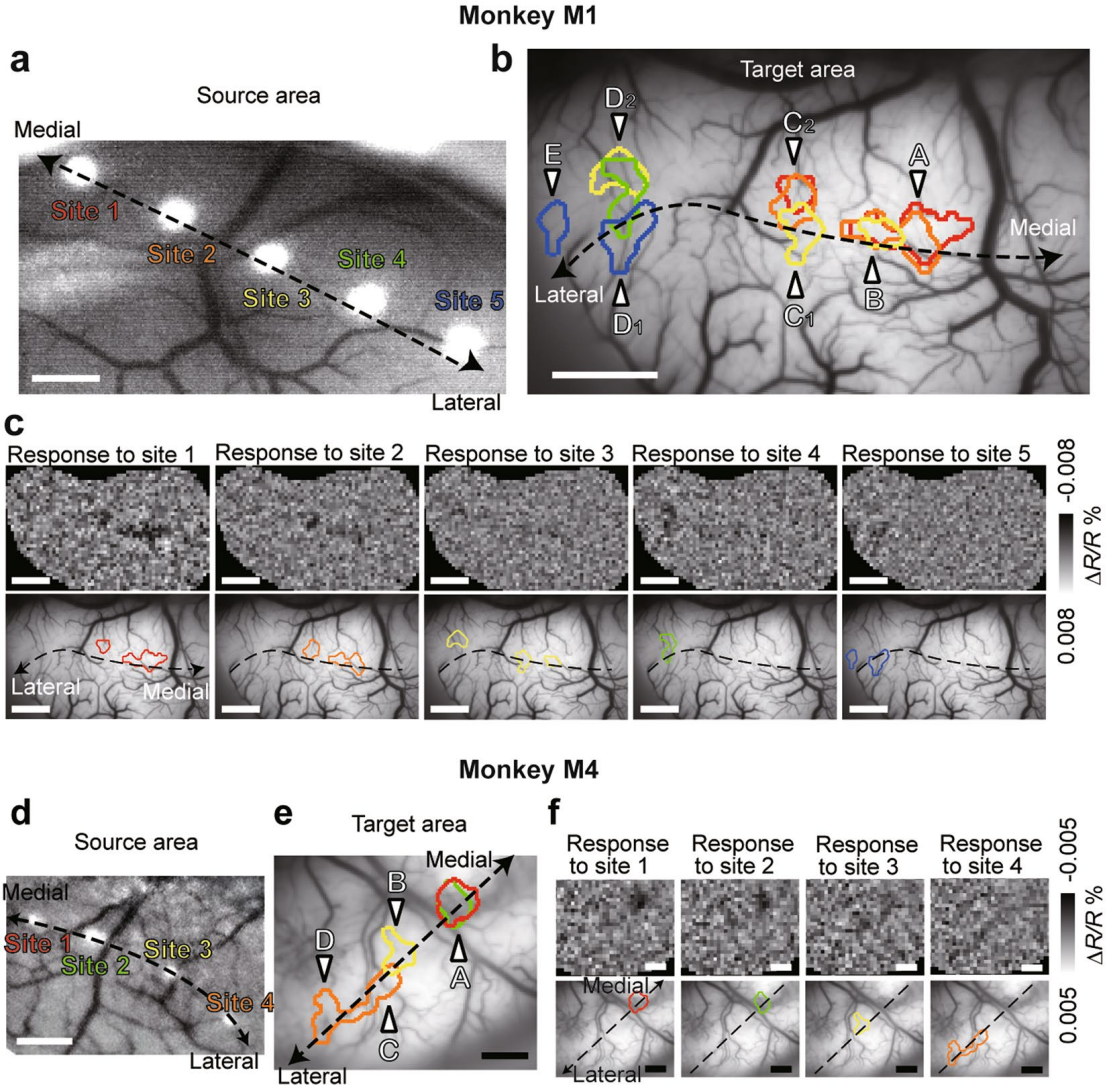
Cortico-cortical projection patterns along V1/V2 borders. (**a**) Optogenetic stimulation sites overlaid on the surface vessel pattern of the source area. Dashed line, the V1/V2 border. (**b**) Activation spots evoked by the stimulation of site 1–5 are overlaid on the vessel pattern of the target area. Color of the contours corresponds to the color of stimulation sites in (**a**). The individual spots are identified by capital alphabets. (**c**) Activation patterns and spots for individual stimulation sites. These results were obtained from M1. Please note that the activation of site 5 was electrophysiologically confirmed in Fig. 4. (**d**–**f**) Cortico-cortical projection patterns obtained from a different monkey (M4). Conventions are the same as in (**a**–**c**). Scale bars in (**a**) and (**d**–**f**), 1 mm. Scale bars in (**b**) and (**c**), 2 mm.

We next explored whether the connections along the V1/V2 borders are mirror symmetric with respect to the midline, i.e., whether these connections are retinotopic (Fig. 6). We did this by stimulating five successive sites at a regular spatial interval along the V1/V2 border in the source area (right hemisphere) in M1 (Fig. 6a). These stimuli evoked seven discrete localized activation spots along the V1/V2 border regions in the target area (left hemisphere; Fig. 6b). The overall projection pattern was retinotopic. The most medial spot A was activated by the stimulation of the medial sites 1 and 2 in the source area. Next to spot A, spots B, C_1_, and C_2_ were activated by sites 1 and 2 but also by site 3 which is next to sites 1 and 2. Spot D_2_ was activated by site 3 but also by site 4 which is next to site 3. Spot D_1_ was activated by site 4 and site 5, the most lateral stimulation site. Finally, spot E was activated exclusively by the stimulation of site 5. The mirror symmetric nature of the projection pattern was also observed in another animal, M4 (Fig. 6d,e). A novel finding revealed by opto-OISI is the existence of both convergent and divergent patterns in interhemispheric connections. In the case of M1, sites 1 and 2 evoked activation at spots A, B, and C_2_, indicating existence of divergent projections from source to target areas. Site 3 evoked activation at spots B, C_1_, and D_2_ (Fig. 6c). Therefore, spot B received convergent inputs from sites 1, 2, and 3, and spot C_2_ received convergent inputs from sites 1 and 2. Similarly in M4, spot A received convergent inputs from sites 1 and 2 (Fig. 6f).

## Discussion

In this study, we proposed a novel method, combining OISI with optogenetics (opto-OISI), to map cortico-cortical projection patterns. We applied this method for visualization of interhemispheric connections around V1/V2 border regions in macaques. The observed projection patterns were reproducible (Fig. 3), and the results were validated through confirmation with MUA recordings (Figs 4 and 5).

The projection patterns were characterized by three features. First, a columnar size activation of neurons by optogenetic stimulation in one hemisphere elicited discrete columnar size activation spots in the other hemisphere (Fig. 3). Second, the interhemispheric projections were restricted to V1/V2 border regions (Fig. 6 and Supplementary Fig. 4). These features are consistent with previous anatomical studies. A study of callosal connections at the V1/V2 border region suggested that the projections are columnar^14^, supporting our current observation of localized activation. This and another study also revealed dense interhemispheric connections at the V1/V2 border region^14,15^. Third, opto-OISI revealed, for the first time, the existence of convergent and divergent interhemispheric connections along the border (Fig. 6). The spatial resolutions of previously available techniques were insufficient to discover these types of interhemispheric connections. To understand the functional and computational roles of these connections, future studies are needed to precisely map the receptive fields and to characterize orientation tuning and ocular dominance properties for connected pairs of sites.

Recently, several proposals have been made for visualizing projection patterns in living monkeys. Ichinohe and colleagues employed fluorescence tracers in living monkeys^21^. They showed that the projection patterns of some fluorescent dyes are detectable at the exposed cortical surface using a fluorescent stereomicroscope. However, this technique depends on the availability of fluorescent dyes, and thus the number of connected pairs that can be identified in a single animal is limited. Functional magnetic resonance imaging (fMRI) combined with optogenetics can be used to visualize area-to-area connections; this method, opto-fMRI, has been used successfully in rodents^22,23^. However, as far as we know, only two reports mentioned opto-fMRI in monkeys^24,25^. Gerits and colleagues showed that optogenetic stimulation in frontal eye field elicited fMRI responses in multiple areas, but locations of activation varied across sessions^24^. Ohayon and colleagues also studied opto-fMRI in the frontal eye field, and showed that fMRI responses were elicited only when optical stimulation was combined with electrical stimulation, suggesting that optogenetic stimulation alone could elicit only sub-threshold fMRI responses^25^. Thus, it is still difficult to combine optogenetics and fMRI in monkey studies. The proposed method, opto-OISI, can overcome these difficulties. There are no limitations in the detectable number of projections, and reproducibility is fairly high.

Previous studies reported difficulty in detecting perturbation by optogenetic stimulation in monkeys. Several studies employed optogenetic stimulation in behavioral studies, and there were no effects or even if there were, the effects were weak^13,24–26^. There are only a few successful cases^27–29^. There are several factors that make OISI with optogenetics possible. First, optogenetic manipulation of neuronal activity with ChR2 largely depends on the expression level of ChR2, specifically the density of ChR2 expression. By inserting the myosin Va binding domain, ChR2 was primarily transported into soma and dendrites, resulting in an increase in the density of ChR2 expression in soma and dendrites without causing cell damage via accumulated viral particles due to multiple infections. A previous report noted that the expression in soma and dendrites was 6 times higher with versus without insertion of the myosin Va binding domain^11^. Second, in contrast to detecting behavior responses to optogenetic stimulation, optical responses were elicited through simple neural circuits between areas: perhaps, monosynaptic connections from one area to another. Finally, in contrast to fMRI, the columnar spatial resolution of OISI enabled us to detect responses of small clusters of neurons. Even if projections evoke sparsely distributed columns, such activation can be detected by OISI but not by fMRI.

In conclusion, we propose that OISI combined with optogenetics is a promising technique to visualize cortico-cortical projection patterns in non-human primates. The method enables us to identify connected pairs in living non-human primates without relying on *a priori* knowledge or assumptions about connectivity. Its application to various cortical areas would increase our understanding of cognitive functions at the neural circuit level in non-human primates.

## Methods

### Animals

Four macaque monkeys (male; *Macaca mulatta*, M1 to M4) were used under anesthesia for all experiments. In two monkeys (M1 and M4), we conducted OISI, extracellular multiunit recordings, and fluorescence imaging experiments. In the other monkeys (M2 and M3), we conducted only fluorescence imaging experiments. The experimental protocol was approved by the Experimental Animal Committee of the RIKEN Institute and followed the guidelines of the RIKEN Institute and the National Institutes of Health.

### Anesthesia

During the initial surgery to attach a head fixation post, the monkeys were initially anesthetized with intramuscular injection of ketamine hydrochloride (5.8 mg/kg), and then deeply anesthetized with intraperitoneal injection of pentobarbital sodium (35 mg/kg). We maintained deep anesthesia by supplemental intravenous injections of pentobarbital sodium (5–10 mg). Rectal temperature was monitored and maintained at 37.6 °C.

During surgery to expose the cortical surface and fluorescence imaging experiments, the monkeys were artificially ventilated with a mixture of N_2_O, O_2_, and isoflurane (70% N_2_O, 30% O_2_, 0.25–2.0% isoflurane). We monitored ECG and EEG and maintained deep anesthesia by adjusting concentration of isoflurane between 0.25 and 2.0%. Expired CO_2_ concentration and rectal temperature were also monitored and were maintained between 3.5 and 4.5% and at 37.6 °C, respectively.

During OISI and extracellular multiunit recording experiments, the monkeys were paralyzed by intravenous injection of vecuronium bromide (67 μg/kg/h) and artificially ventilated with a mixture of N_2_O and O_2_ (70% N_2_O, 30% O_2_). To eliminate a small possibility that monkeys feel pain, fentanyl citrate (0.83 g/kg/h) was infused intravenously and continuously throughout the experiments. EEG, ECG, expired CO_2_ concentration, and rectal temperature were monitored throughout the experiments. Expired CO_2_ and rectal temperature were maintained between 4.0 and 5.0% and at 37.6 °C, respectively.

### Surgical procedures

The surgical procedure involved implantation of a head fixation post and of chambers for optogenetic stimulation and optical imaging. In the initial surgery, we implanted the head fixation post. The head fixation post was attached to the top of the skull. After the attachment, two stainless-steel bolts to record EEG were implanted through the skull above the dural surface of left and right frontal cortices. At a far location from the two bolts, an inverted T-shaped bolt was implanted through the skull for electrical grounding purposes. We attached the flat surface of the inverted T-shaped bolt on the dural surface for stable grounding.

After recovery from the initial surgery, we implanted a recording chamber in the right hemisphere (source area). The center of the chamber approximately positioned to be slightly below the lunate sulcus. After fixing the chamber to the skull with dental cement, the skull and the dura inside the chamber were removed for vector injection. Another chamber was implanted in the left hemisphere (target area) 30–70 days after the vector injection. The exposed cortex was covered with a layer of transparent artificial dura made of silicon rubber. The inside of the chamber was filled with saline for fluorescent imaging or heavy silicon oil (10 St) for OISI, and a glass coverslip was attached to the chamber. For the virus injection or extracellular recordings, the exposed cortex was covered with a transparent artificial dura with a small hole or a slit through which we inserted glass capillaries. During caging, the chamber was filled with 2% agarose with dexamethasone (8.25 μg/ml) and antibiotic (25 μg/ml).

### AAV vectors

AAV vector (serotype 9), AAV9-CaMKIIα-hChR2(ETTC)-EYFP expressed a humanized ChR2 (E123T/T159C)^19^ of which the c-terminal was fused to enhanced yellow fluorescence protein [hChR2(ETTC)-EYFP] under the control of the CaMKIIα promoter (1.3 kb). AAV9-CaMKIIα-hChR2(ETTC)-EYFP was constructed with pAAV-CaMKIIα-hChR2(E123T/T159C)-EYFP^30^ that was a gift from Karl Deisseroth (Addgene plasmid # 35511). AAV vector (serotype9), AAV9-CaMKIIα-hChR2(ETTC)-EYFP-MBD expressed hChR2(ETTC)-EYFP of which the c-terminal was fused to MBD [hChR2(ETTC)-EYFP-MBD] under the control of the CaMKIIα promoter (1.3 kb). AAV9-CaMKIIα-hChR2(ETTC)-EYFP-MBD was constructed with pAAV-CaMKIIα-hChR2(E123T/T159C)-EYFP-MBD. pAAV-CaMKIIα-hChR2(E123T/T159C)-EYFP-MBD was constructed as follows. DNA fragment of myosin Va binding domain of melanophilin (MBD; 113 bp) was obtained by *Xho*I digestion of CHR2-MBD^11^ that was a gift from Don Arnold (Addgene plasmid # 21484). The *Xho*I fragment blunted by DNA polymerase was inserted into the blunt-end *Eco*RI site of pAAV-CaMKIIα-hChR2(E123T/T159C)-EYFP. The direction of insertion of MBD was confirmed by *Eco*T22I digestion. The original stop codon (TAA) of pAAV-CaMKIIα-hChR2(E123T/T159C)-EYFP was changed to GGC by PCR with PrimeSTAR^TM^ HS DNA polymerase (#R010Q, TAKARA Bio) and primers (5′-TAC AAG GGC GAA TTT CGA GAT CAA CCA-3′; 5′-AAA TTC GCC CTT GTA CAG CTC GTC CAT-3′). The packaging, purification, and titration of the AAV vectors (serotype 9) was performed by SignaGen® Laboratories (Rockville, MD, USA). The AAV9 vector had a titer of 10^13^ particles/ml.

To test feasibility of the proposed method (OISI combined with optogenetic stimulation), we used AAV vector, AAV9-CaMKIIα-hChR2(ETTC)-EYFP-MBD. We also used vectors, AAV9-CaMKIIα-hChR2(ETTC)-EYFP, as a control to examine specificity of ChR2 expression with and without insertion of MBD (Supplementary Fig. 1). We performed pressure injection of each AAV vector (0.25–2 μl) with a glass capillary at 0.4 mm, 0.8 mm, and 1.2 mm of the depth from cortical surface in following steps: (1) we penetrated the glass capillary to 1.6 mm depth from the cortical surface, (2) retracted the electrode for 0.4 mm and made a pressure injection of the vector, and (3) waited for 1–3 min for the vector to diffuse into the trajectory of the glass capillary. In addition to the injection at 1.2 mm depth, we repeated steps (2) and (3) for two more times to make injections at the depth of 0.4 and 0.8 mm.

### Fluorescence imaging

We used a fluorescence microscope (Keyence, VB-7000; 1360 × 1024 pixels, 24-bits color) to monitor fluorescence intensity of the reporter protein (EYFP) at the cortical surface. We illuminated the cortical surface by the light at excitation wavelength of EYFP (470 ± 40 nm), and captured the cortical surface image at the fluorescence emission wavelength through a bandpass filter (535 ± 50 nm; exposure time, 5 s) with the microscope (image *F*; Supplementary Fig. 2b). The captured fluorescence image of EYFP did not directly reflect the spatial pattern of EYFP expression because of uneven illumination. To compensate spatially uneven illumination of the excitation light, the excitation light image (image *E*; Supplementary Fig. 2a) was also captured without using the bandpass filter (exposure time, 50 ms), and calculated ratio of gray scaled images of *F* and *E* (image *F*/*E*; Supplementary Fig. 2c). Strictly speaking, image *E* was generated not only by reflected light at the excitation wavelength but also by fluorescence elicited by the excitation light. However, the fluorescence component in image *E* was negligibly small compared with the excitation light component. To obtain time courses, a histogram of the ratio image *F/E* was calculated over the area of AAV9-CaMKIIα-hChR2(ETTC)-EYFP-MBD injection for each of fluorescence monitoring days. We fitted the histogram with a Gaussian function (Supplementary Fig. 2c,d), and its mean value and the standard deviation was used to plot the time courses of ChR2 expression (Supplementary Fig. 2e).

### Optogenetic light stimulation

We used custom-made optical apparatus (Olympus) to precisely adjust locations of the focal light stimulation relative to the cortical surface vessel pattern (Fig. 1a, optogenetic stimulation side). The apparatus was equipped with an X-Y motorized stage to scan the stimulation sites and a CCD camera to monitor both the cortical surface and the spot of light stimulation. The spot size was 100 μm at the focal point. The light was focused to deeper layers since there are few neurons are in layer I. We used high-power laser light source (Lucir, COME 2 Series) for the light stimulation at 473 nm. With this laser light source, estimated power of light at the cortical surface reached up to 34 mW. Pulsed light was used for the light stimulation. The frequencies were 5, 10, or 20 Hz for OISI and 1 to 20 Hz for extracellular multiunit recordings. The durations were 7 s for OISI, 1 s (Fig. 5) and 6.4 s (Fig. 4) for extracellular multiunit recordings. In OISI, recordings were repeated 48–96 trials. Each trial included multiple site stimulation with different pulse frequencies. The order of stimulation sites and frequencies was randomized within a trial. In extracellular multiunit recordings, a recording session for a stimulation site included 32–64 trials, and each trial consisted of recordings with different pulse frequencies. The order of stimulus frequencies was randomized within a trial. In Fig. 5, the stimulation site was changed from session to session to scan 12 different sites. In OISI and extracellular multiunit recordings, the source area was covered with a transparent artificial dura to protect from drying.

### OISI recordings

We used OISI to detect two-dimensional activation patterns in the target area. The exposed cortex was illuminated with 600 nm light. The reflected light from the cortex was detected with a CCD camera (Sony, SC-7500) and the video signals were digitized by a 10-bits video capture board (Matrox, Corona-II). We used a neutral density filter that compensates uneven illumination of the cortex^31^ and made brightness of the cortex spatially homogeneous before the CCD camera captured the cortical image (Fig. 1a, recording side). The filter was used in M1 but not in M4. The imaging area was 13.4 × 10.1 mm (320 × 240 pixels). The onset of the video signal acquisition was synchronized to the inhalation phase of the artificial ventilator, and the onset of light pulse stimulation was set to 1 s after the onset of the video signal acquisition. The video signals were continuously acquired for 8 s with a frame rate of 0.5 s. To identify activation area on the cortical surface, the vessel patterns were captured with 540 nm light before OISI recording.

### OISI data analysis

The acquired images in OISI recordings contained stimulus independent noises, such as spontaneous fluctuation of hemodynamic signals and surface reflection changes due to respiration and heartbeat. Signal-to-noise ratio between specific modulation elicited by optogenetic stimulation and the other components may not be large. To reliably extract the stimulus dependent component from the noisy image, we first preprocessed OISI data in the conventional way^7,32^: (1) differential image *ΔR*/*R* was calculated by dividing the averaged image obtained between 0.5 and 8 s after the stimulus onset by those obtained during a 1 s period before the stimulus onset, (2) we set a region of interest on V1/V2 border regions, (3) error trials, e.g. existence of bubbles running across the recording area, were removed, and (4) we applied spatial binning (4 × 4 pixel). Then, we extracted activation patterns for each stimulus by calculating a generalized indicator function proposed by Yokoo and colleagues^33^. The generalized indicator function provides reliable condition-specific responses by maximizing a weighted difference between signal and noise variances, and the signal-to-noise ratios satisfy user-defined level of significance. We set the level of significance (signal-to-noise ratio) to 4. We defined activation spot that is a cluster of the pixels (>0.11 mm^2^) with 2.5 times larger darkening than mean response in the activation map, and outlined by connecting pixels with half the peak darkening in the activation map filtered with Gaussian (*σ* = 168 μm).

### Extracellular multiunit recordings

For the experiments in M1, we used an electrode array with 8 shanks each of which consisted of 8 electrical contacts with 200 μm spacing (NeuroNexus, A64). The shank to shank distance was also 200 μm. For the experiments in M4, we used an electrode having 16 electrical contacts with 150 μm spacing (Plexon, V-probe). We penetrated electrodes perpendicularly to the cortical surface and advanced them until neuronal activities were observed from all electrical contacts. The raw electrical signals were amplified and bandpass filtered (500 to 3000 Hz) with a preamplifier (Tucker Davis Technologies, RZ2). The filtered signals were digitized at 25 kHz and stored in a computer. We analyzed spike rates of multiunit activity (MUA) obtained by the time stamp when filtered signal exceeded a fixed threshold (3.5 times the standard deviation of background noise).

### MUA data analysis

We first recorded MUA responses to visual stimuli of moving gratings to find reliable electrical contacts, and then recorded responses to optogenetic light stimulation. We excluded the electrical contacts from further analyses if the visual responses were not statistically significantly different from spontaneous activities (two-tailed t-test, significance level = 0.001, *N* = 8 stimuli) and orientation sensitivity index was less than 0.4; the electrical contacts of 9 among 56 for data in Fig. 4, one among 16 for data in Fig. 5b, and one among 16 for data in Fig. 5c were excluded, respectively. In the analyses of the responses to optogenetic light stimulation, we calculated PSTHs for each stimulus with a bin size of 10 ms. We observed modulation of PSTHs with optogenetic stimulation primarily in superficial layers (0 to 1.2 mm from cortical surface) as suggested by a previous anatomical study^14^. In Fig. 5, we averaged the PSTHs of the electrical contacts down to the depth where stimulus specific modulation was observed. In Fig. 4, we averaged the PSTHs within a shank down to the depth where stimulus specific modulation was observed in any of the electrical contacts across eight shanks. We applied Fourier analysis to the averaged PSTHs in Fig. 4c–f. In Fig. 5b,c, we applied two-tailed paired t-test to mean firing rates in pre-stimulus period (−0.5–0 s from stimulus onset) and in light irradiation period (0–0.25 s and 0.5–0.75 s) to test whether modulation by the light stimulation was significant or not (significance level = 0.001, *N* = 32 trials).

## Supplementary information

Supplementary Figures
