# Supplementary material for: Optical intrinsic signal imaging with optogenetics reveals functional cortico-cortical connectivity at the columnar level in living macaques
Source: Sci Rep. 2019 Apr 23;9:6466. doi: 10.1038/s41598-019-42923-2 (PMC6478906; doi:10.1038/s41598-019-42923-2)
Supplement: Supplementary file 1 — Supplementary Figures [file 41598_2019_42923_MOESM1_ESM.pdf]

Supplementary Information

Optical intrinsic signal imaging with optogenetics reveals functional cortico-cortical connectivity at the columnar level in living macaques

Yu Nakamichi[1], Kai Okubo[1,2], Takayuki Sato[1], Mitsuhiro Hashimoto[3], Manabu Tanifuji[1,2,4*]

1. Lab for Integrative Neural Systems, RIKEN Center for Brain Science, Wako, Saitama 351-0198, Japan.
2. Department of Complexity Science and Engineering, Graduate School of Frontier Sciences, The University of Tokyo, Kashiwa, Chiba 277-8561, Japan.
3. Department of Neuroanatomy and Embryology, Fukushima Medical University, Fukushima, Fukushima, 960-1295, Japan.
4. Department of Life Science and Medicine, Bio-Science Faculty of Science and Engineering, Waseda University, Shinjuku, Tokyo 169-8555, Japan.

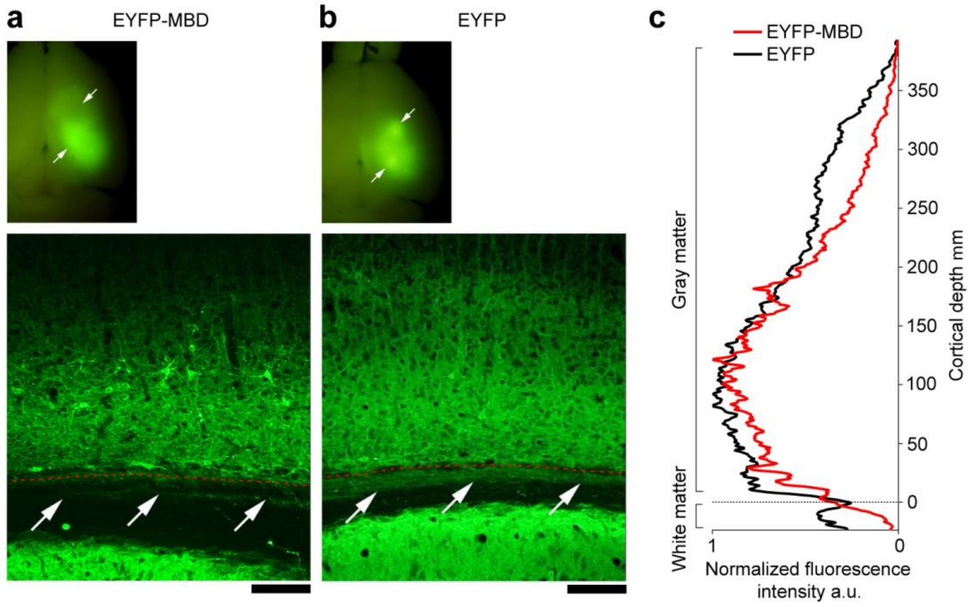

Supplementary Fig.1. Localization of ChR2 expression to dendrite and cell soma by the addition of myosin Va binding domain (MBD).

(a,b) Fluorescence images of the mouse brain injected with AAV9-CaMKIIα-hChR2(ETTC)-EYFP-MBD (a) and with AAV9-CaMKIIα-hChR2(ETTC)-EYFP (b). Upper panels, the virus injection sites of each vector (arrows). Lower panels, the coronal sections cut from the posterior injection sites in the upper panels. Red dashed lines, the border between gray and white matters. Arrows, location of the white matter. Scale bar, 100 μm. (c) Spatial profiles of the fluorescence intensity along the depth axis for **a** (red trace) and for **b** (black trace). The florescence intensity (horizontal axis) represents averaged fluorescence at the same distance from the border between gray and white matters that is normalized into 0 (minimum) to 1 (maximum). As observed in the arrows in the lower panels of **a** and **b**, there was clear difference in fluorescence in the white matter, suggesting lack of ChR2 in axons by the addition of MBD to the c-terminal of hChR2(ETTC)-EYFP. The injection volume of the AAV vectors was 0.5 μl and the survival period was 12 days. In both **a** and **b**, we put the center of the field of view of the microscope at the deeper layers to reliably visualize difference in fluorescence around the white matter. Additional difference in fluorescence seen at superficial layers could be also due to the lack of ChR2 in axons with MBD but the observation is not convincing to us because these layers were placed at the peripheral field of view.

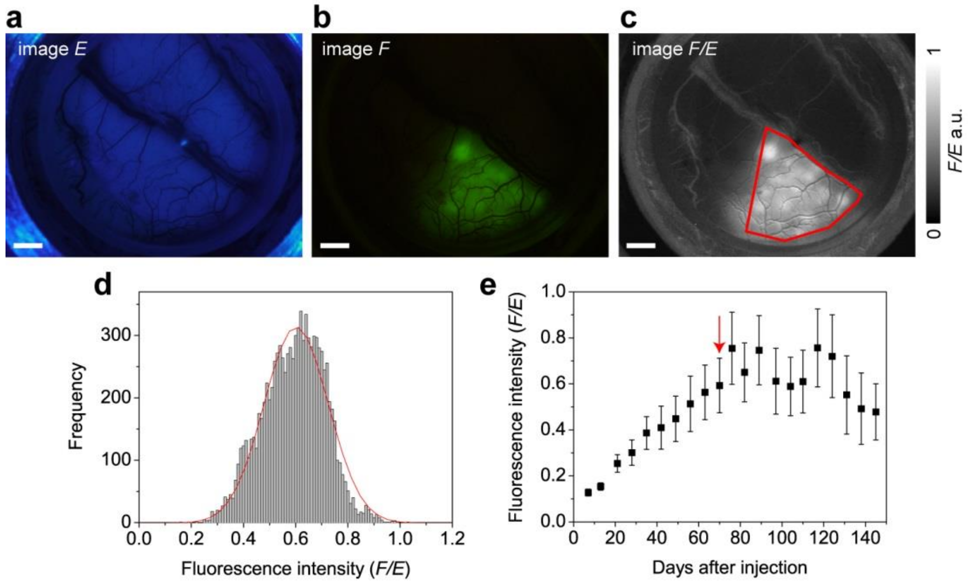

Supplementary Fig. 2. Quantification of fluorescence intensity.

(a) Cortical image with excitation light irradiation (image *E*). In this and also in other cases, the exposure time was fixed to 50 ms. (b) EYFP fluorescence image of the cortex (image *F*). In this and also in other cases, the exposure time was fixed to 5 s. (c) Ratio image between the images of fluorescence and excitation light (image *F*/*E*). The region enclosed by red line is the area of vector injection sites. Scale bars, 2 mm. (d) Distribution of pixel values of image *F*/*E* for the enclosed region in **c**. The red trace gives a fitting curve assuming a Gaussian distribution. (e) Time course of ChR2 expression (quantified fluorescence intensity). The symbols represent mean and standard deviation of distributions. Red arrow, the case of **d**. The data were obtained from M2.

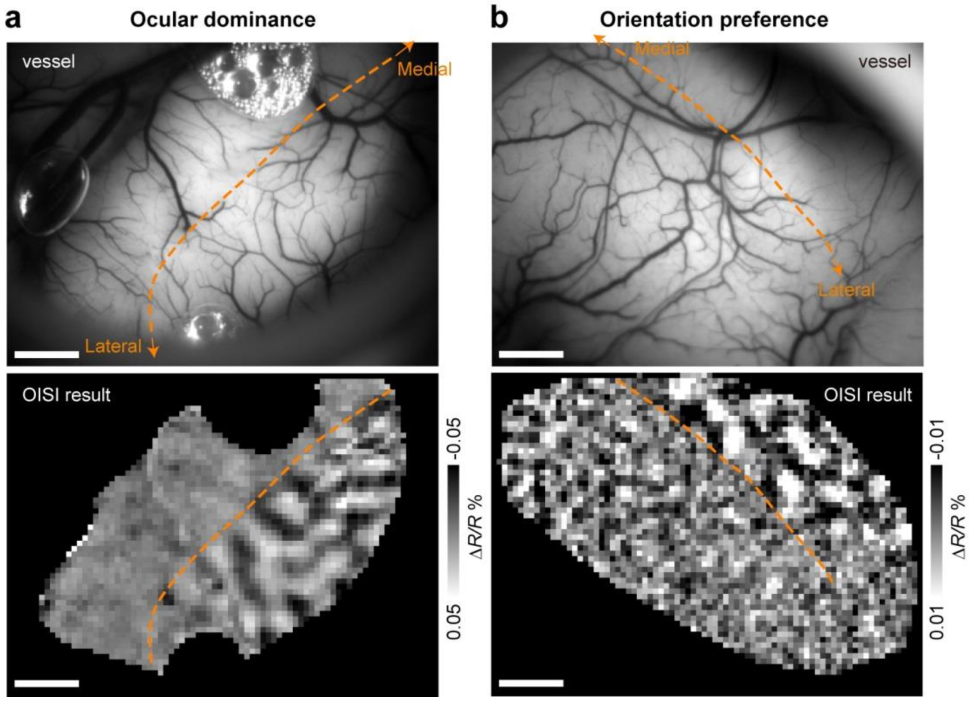

Supplementary Fig. 3. Ocular dominance and orientation preference maps obtained by OISI to detect V1/V2 border.

(a) Vessel patterns (upper panel) and ocular dominance column (lower panel) in left V1/V2 border region. We separately presented 0, 45, 90, and 135 deg moving grating patterns to right and left eyes and calculated a differential map between responses to right and left eye stimulations. Since ocular dominance columns are obscured in V2, we defined V1/V2 border as the line where ocular dominance columns were terminated (dashed orange lines). (b) Vessel patterns (upper panel) and differential map between responses to 45 and 135 deg moving grating patterns (lower panel) in right V1/V2 border region. Since V2 neurons have larger receptive fields than V1 neurons, the size of patches obtained in differential map differs among V1 and V2. We defined V1/V2 border as the line where size of the patches changed (orange lines). Scale bars, 2mm. The data were obtained from M1.

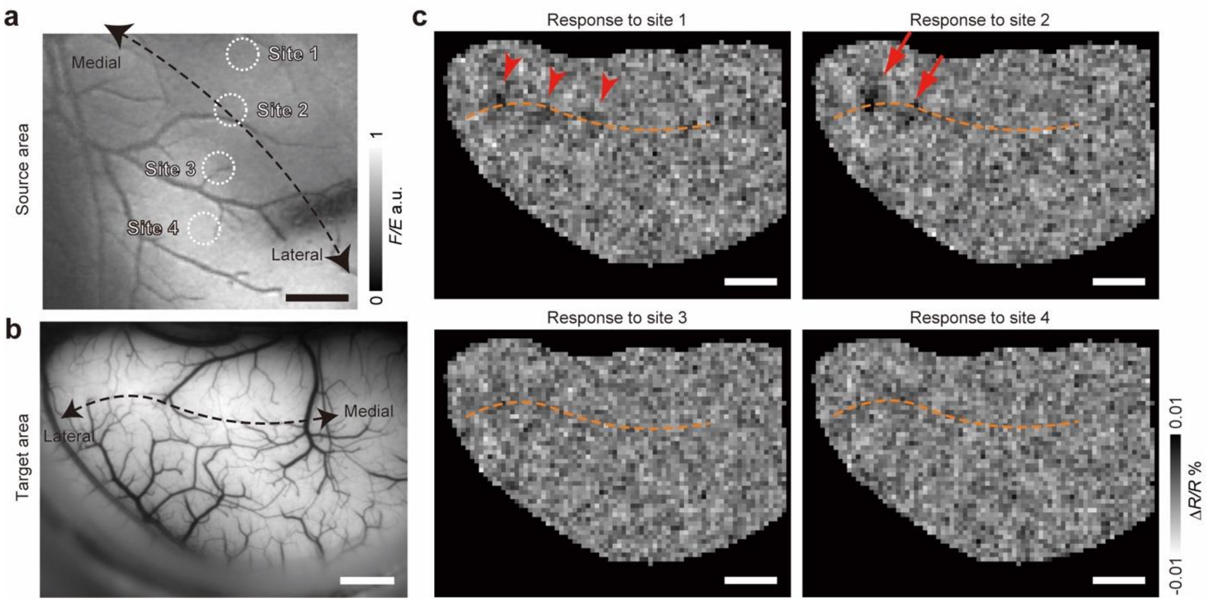

Supplementary Fig. 4. Activation maps elicited by focal light stimulation at various locations around V1/V2 border.

(a) Fluorescence image of EYFP (*F/E*) in the source area (right hemisphere). White dashed circles indicate focal light stimulation sites. (b) Vessel pattern of the target area (left hemisphere). (c) Activation maps in the target area obtained by stimulation of each site. Site 2 stimulation elicited two activation spots (upper-right panel, arrows). We observed weak activation spots along the border by stimulation of site 1 (upper-left panel, arrowheads). As shown in **a**, site 1 located 1 mm apart from the V1/V2 border in V2. This result is consistent with widely but sparsely distributed callosal connections in V2 in a previous report[15]. Stimulation at sites 3 and 4 located in V1 essentially evoked no activation in the target area (lower panels in **c**). These observations revealed that the interhemispheric connection was restricted at least to the region about 1 mm from the V1/V2 border. Scale bar in **a**, 1 mm. Scale bars in **b** and **c**, 2 mm. Dashed line, V1/V2 border.
